# The Relationship between Low Serum Vitamin D Levels and Altered Intestinal Barrier Function in Patients with IBS Diarrhoea Undergoing a Long-Term Low-FODMAP Diet: Novel Observations from a Clinical Trial

**DOI:** 10.3390/nu13031011

**Published:** 2021-03-21

**Authors:** Michele Linsalata, Giuseppe Riezzo, Antonella Orlando, Benedetta D’Attoma, Laura Prospero, Valeria Tutino, Maria Notarnicola, Francesco Russo

**Affiliations:** 1 Laboratory of Nutritional Pathophysiology, National Institute of Gastroenterology “S. de Bellis” Research Hospital, 70013 Castellana Grotte, Ba, Italy; michele.linsalata@irccsdebellis.it (M.L.); giuseppe.riezzo@irccsdebellis.it (G.R.); antonella.orlando@irccsdebellis.it (A.O.); benedetta.dattoma@irccsdebellis.it (B.D.); lauraprospero87@gmail.com (L.P.); 2 Laboratory of Nutritional Biochemistry, National Institute of Gastroenterology “S. de Bellis” Research Hospital, 70013 Castellana Grotte, Ba, Italy; valeria.tutino@irccsdebellis.it (V.T.); maria.notarnicola@irccsdebellis.it (M.N.)

**Keywords:** intestinal barrier, irritable bowel syndrome, low FODMAP diet, symptom profile, vitamin D

## Abstract

Decreased serum vitamin D (VD) levels have been associated with gastrointestinal (GI) disorders, including irritable bowel syndrome (IBS). VD can also modulate the intestinal barrier. Given the link between the GI barrier’s alterations and diet, attention has aroused the positive effects of the Low FODMAP Diet (LFD) on IBS patients’ symptom profile. We evaluated the GI symptoms and the urinary and circulating markers of GI barrier function, the markers of inflammation and intestinal dysbiosis in 36 IBS patients with predominant diarrhea (IBS-D) (5 men and 31 women, 43.1 ± 1.7 years) categorized for their circulating VD levels in low (L-VD) and normal (N-VD) (cutoff = 20 ng/mL). Evaluations were performed before and after 12 weeks of LFD. At the baseline, L-VD patients showed a significantly worse symptom profile and altered small intestinal permeability (s-IP) than N-VD. After LFD, a significant increase in the circulating VD levels in both the subgroups and a significant improvement of s-IP in L-VD patients occurred. Finally, VD levels negatively correlated with the symptom score and fecal zonulin. These data highlight the close relationship between VD and the intestinal barrier and support their involvement in IBS-D pathophysiology. Moreover, the potentially positive role of LFD in the management of IBS-D was confirmed.

## 1. Introduction

Vitamin D (VD) belongs to the steroid hormone family, and evidence is accumulating on its influence on human health homeostasis [1]. VD is effective as an immune modulator, anti-inflammatory, and antimicrobial agent [2], and its deficiency has recently been linked to different disorders of the gastrointestinal (GI) tract, including intestinal motility disorders (e.g., functional constipation) [3] and irritable bowel syndrome (IBS) [4,5].

The major circulating form of VD is 25-hydroxy VD [25(OH)D], which is also used to assess VD status in clinical practice [6]. There is no universally accepted optimal level of VD. However, a North American expert board stated that serum levels of 25(OH)D must exceed 20 ng/mL to be adequate to meet the needs of 97.5% of the population. Consequently, levels lower than 20 ng/mL must be considered insufficient [7]. In line with this conclusion, an extensive revision of VD levels in a Mediterranean population has recently established a cutoff level of at least 20 ng/mL that could better reflect the normal physiology in our geographic areas [8].

A considerable percentage of the IBS population can be VD deficient, and, recently, the successful treatment of diarrhea-predominant IBS (IBS-D) and its associated symptoms, with high oral doses VD supplementation, has aroused the attention for this hormone as a therapeutic option for the IBS-D management [9]. Intolerance to dairy or fatty acids and altered digestive patterns may be responsible for VD deficiency, considering that 20% of the vitamin derives from dietary sources and absorbed with fat in the small bowel [10]. Therefore, it is also conceivable that an impaired small intestinal absorption capacity consequent to various GI diseases could be one of the possible factors related to the onset of VD deficiency [11].

VD also has shown potentialities in modulating the intestinal barrier [12], and there is a growing body of evidence for its protective effects on the epithelial barrier in the gut mucosa, suggesting implications for the “leaky gut” onset and changes in the small intestinal permeability (s-IP) [13]. This aspect is critical since it is now accepted that a dysfunctional intestinal barrier, mainly in the upper gut, could be the origin or consequence of persistent low-grade immune activation and may play an essential role in IBS-D pathogenesis [14]. Altered gut permeability allows the passage of the luminal contents into the bloodstream, resulting in the activation of the immune response and gut inflammation induction [15]. The evaluation of s-IP is based on functional urinary tests that use non-absorbable sugars of different sizes, such as lactulose (La), mannitol (Ma), and sucrose (Su) [16]. La is a disaccharide that provides information on the paracellular pathway and tight junction (TJ) integrity, while Ma is a monosaccharide, and it is thought to reflect the transcellular route. Hence, in clinical practice, the urinary La/Ma ratio is used as a reliable parameter to evaluate the impairment of s-IP. Finally, Su is an index of gastroduodenal permeability [17].

Other tests to evaluate the GI barrier function include the fecal and serum levels of zonulin, a human protein that reversibly regulates s-IP by changing the TJ interaction [18]. Additionally, the intestinal fatty acid-binding protein (I-FABP) [19] and diamine oxidase (DAO) [20] are now considered as potential markers for the intestinal epithelial barrier health since they are immediately released in response to the cell membrane’s altered integrity and subsequently appear in the bloodstream [21].

Many IBS patients often describe a worsened symptom profile in consuming certain foods, such as those containing the so-called fermentable oligosaccharides, disaccharides, monosaccharides, and polyols (FODMAP) [22]. Consequently, a low FODMAP diet (LFD) has been proven useful for treating IBS-D. In a recent study, IBS-D patients largely benefited from a long-lasting LFD in terms of symptoms by improving at the same time their inflammatory and lipidomic profiles [23].

Based on these premises, our research was designed to investigate the relationship between VD, evaluated as 25(OH)D, s-IP, and the integrity of the GI barrier in IBS-D patients following a long-term (12 weeks) LFD. The main aim of this study was to compare the GI symptoms, assessed by the IBS symptom severity scale (IBS-SSS) [24] and the above-mentioned urinary and circulating markers of intestinal barrier function and integrity in IBS-D patients with VD deficiency (L-VD) compared to those with normal VD levels (N-VD), before and after LFD. The minimal inflammation in IBS-D was assessed by dosing interleukins 6 and 8 (IL-6 and IL-8). Lastly, lipopolysaccharide (LPS) and the urinary markers of intestinal dysbiosis (indican and skatole) were also evaluated.

## 2. Materials and Methods

### 2.1. Patient Recruitment

IBS-D patients, diagnosed according to the Rome IV criteria, were recruited from January 2018 to September 2019, among the outpatients of the Nutritional Physiopathology Laboratory—National Institute of Gastroenterology “S. de Bellis” Research Hospital located in Apulia, a Mediterranean region in south-eastern Italy.

After a physical examination, patients completed the “Gastrointestinal Symptom Rating Scale” (GSRS) questionnaire [25], provided a basal urinary sample, and underwent a blood withdrawal for complete blood count, liver and thyroid function tests, and inflammatory markers. Eligibility was determined by gastroscopy, colonoscopy, fecal occult blood test (3 determinations), stool culture, stool ova and parasites tests.

The female patients provided the blood and urinary samples during the follicular phase (within ten days of the onset of the most recent menstrual cycle) to avoid interference and contamination of the urine samples with blood. The inclusion criteria were age 18 years or older; symptoms resembling IBS-D for at least 14 days; a stool pattern, as described by Schmulson et al. [26]; a minimum average of 3.0 on the seven-point Likert scale of the GSRS; no restriction on eating and drinking. Anti-endomysium and tissue transglutaminase antibodies had to be negative. Moreover, only the IBS-D patients resulting HLA-DQ2/HLADQ8 negative/negative were included to avoid the occurrence of symptoms due to non-celiac gluten-sensitivity (NCGS) observed in some IBS patients with the presence of HLADQ2 and/or DQ8 [27].

The exclusion criteria were: pregnancy; intense physical activity; constipation; fever; a diagnosis of post-infectious IBS, giardiasis, previous abdominal surgery, endocrine and metabolic disorders; cardiovascular diseases; altered hepatic and renal functions; secondary causes of intestinal atrophy, previous diagnosis of neoplasms; the use of drugs for alleviating IBS symptoms or probiotics in the two weeks before evaluation; previous antibiotic therapy; the use of selective serotonin reuptake inhibitors or other antidepressant drugs. The reasons for study interruption (e.g., lack of follow-up, specified adverse events, death, and other causes) were recorded in the case report form. Written informed consent was obtained from all the subjects to collect anthropometric values, clinical data, and analytical measurements.

To avoid possible bias due to the exposure to sunlight, the enrollment of patients was suspended during the summer months.

This study was part of a research project approved by the local Scientific Committee and the Institutional Ethics Committee of IRCCS Ospedale Oncologico—Istituto Tumori Giovanni Paolo II, Bari, Italy, (N. 274/C.E. 12.12.17), and it was part of a registered clinical trial on http://www.clinicaltrials.gov (NCT03423069)-last accessed data: 17 February 2021.

### 2.2. Study Design

The study design included three visits.

Visit 1 (Baseline). The subjects underwent a gastroenterological examination and received verbal and written information about the study. They gave their informed consent about the goals of this study to evaluate the efficacy of a diet intended to reduce IBS symptoms and be followed for 12 weeks. Then, subjects underwent an interview with trained nutritionists to obtain information on their lifestyle, physical activity, dietary habits, physiological and pathological conditions. The eligible patients were invited to consume their usual diet and fill in a daily diary of their food habits for the following seven days. The diary included the record of the stool characteristics based on the intestinal habits, the Bristol stool form chart [28], the use of medications, physical activity, and food habits to estimate the daily energy intake and energy consumption.

Visit 2 (Diet attribution). One week after visit 1, the anthropometric measurements were performed. All the patients completed the IBS-SSS questionnaire [24]. They had to get a total IBS-SSS score >125 to be recruited in the study. The inclusion and exclusion criteria were reconsidered, including eating habits, by evaluating the daily food diary completed in the week preceding the visit. Patients received their personalized diet and were invited to fill a daily diary until the end of the diet. Moreover, they had to record food, intestinal habit, physical activity, medications, and stool characteristics according to the Bristol stool form chart [28]. All the patients carried a stool and urine sample and underwent a blood sample withdrawal for the analytical measurements and the sugar absorption test (SAT).

Visit 3 (final visit). After 12 weeks of the diet, the researchers collected the symptoms and food questionnaires filled in the previous days. The patients received the IBS-SSS and a food diary for checking adherence to the diet (IBS diet-adherence report scale—IDARS). All the patients underwent the same procedures again as at visit 2 for the anthropometric measurements and analytical measurements.

### 2.3. Symptom Profile

The IBS-SSS, a validated GI symptoms questionnaire [24], was used to assess the symptom profile. This questionnaire provides a global measure of the severity of IBS symptoms by evaluating five items on a visual analog scale (VAS). The five items included: (1) “severity of abdominal pain”, (2) “frequency of abdominal pain”, (3) “severity of abdominal distension”, (4) “dissatisfaction with bowel habits”, (5) “impact of symptoms on quality of life”. Each symptom was described on a 100-point scale. For (1) to (4) items, patients marked a point on the line that reflected how they felt, and the proportional distance from zero was the score (ranging from 0 to 100) assigned for that item. The final item (5) required the number of days out of ten during which the subjects complained of “abdominal pain”. The answer was multiplied by 10 to create a metric scale from 0 to 100. The five items were summed, providing a total score ranging from 0 to 500. Scores identified “mild” (75 to 175),”moderate” (175 to 300), and “severe” cases (>300). Conventionally, healthy subjects have a score below 75, and patients with scores lower than 75 should be considered in a remission phase.

### 2.4. Assessment of Nutrient Intake

The patients had to record a food diary at the start of the study and during the diet intervention to evaluate their energy intake and energy consumption. The diary included details of the quantities (expressed in grams) and the types of food consumed daily at breakfast, lunch, dinner, during snacks, and the type of physical activity and duration [23].

Nutritionists evaluated food diaries completed before and during the diet period. All data were analyzed by dedicated software (Progetto Dieta v. 2.0—http://www.progettodieta.it-last accessed data: 18 March 2020). The daily energy intake and consumption expressed in kcal, the percentage and weight of daily carbohydrates, lipids, proteins, the percentage of alcohol, the weight of the dietary fiber, minerals, and vitamins, were calculated.

### 2.5. Intervention Diet

A personalized LFD was assigned after examining the food diaries and face-to-face individual counseling with the nutritionists at visit 2. LFD implies a restricted intake of FODMAPs [29]. A dedicated software (Nutrigeo 8.6.0.0, Progeo Medical, Centobuchi di Monteprandone, AP, Italy) was used to evaluate the daily macronutrient intake (50% glucides, 30% lipids, and 20% proteins). The diets were developed as described elsewhere [23]. A detailed weekly menu structured on breakfast, lunch, and dinner, plus two light snacks (mid-morning and afternoon) was provided to each patient together with a brochure reporting detailed information on the permitted and forbidden foods, and which to reduce according to the indications of the Monash University [30]. An adequate fiber intake was guaranteed, and alcohol intake was not recommended. In-between visits were performed every 30 days, during which patients had to fill the IDARS.

### 2.6. Sugar Absorption Test

All the participants in the study underwent s-IP evaluation by SAT after fasting overnight. Pretest urine was collected in our laboratory to check for the possible presence of endogenous sugars. Then subjects drank a sugar test solution containing 10 g of La, 5 g of Ma, and 40 g of Su in a volume of 100 mL. Urine samples were collected up to 5 h after administration. A 1 mL volume of 20% (*w/v*) chlorohexidine was added to each collection as a preservative regardless of the final volumes. The total urine volumes from individuals were measured and recorded. After thoroughly mixing, a portion of 2 mL was taken and stored at −80 °C until examined. The detection and measurement of the three sugar probes, La, Ma, and Su in urine, were performed by chromatographic analysis, as described previously by our group [31]. The percentage of ingested La (% La), Ma (% Ma), and Su (% Su) were evaluated, and the La/Ma ratio was calculated for each sample. Patients with a La/Ma ratio higher than 0.030 were considered as having an altered s-IP [32].

### 2.7. Biochemical Assays

The biochemical evaluations were performed at enrollment and the end of the diet. After 12 h of fasting, a blind coded sample of whole blood was taken from each IBS-D patient by venipuncture and collected in vacutainer tubes containing ethylene–diamine–tetra-acetic acid (EDTA-K2) anticoagulant. Raw stool samples from the IBS-D patients were frozen and stored at −80 °C within 12 h after the sampling. Before the laboratory analysis, stool samples were thawed, and mechanical homogenization was performed using an inoculation loop. The Fecal Sample Preparation kit (Immunodiagnostik AG, Bensheim, Germany) to prepare fecal eluates was used. VD status was quantitatively assessed by measuring serum 25(OH)D levels using a chemiluminescence system (DiaSorin, Stillwater, MN, USA). Serum and fecal zonulin were assayed by ELISA kits (Immunodiagnostik AG, Bensheim, Germany). Following the manufacturer’s instructions, serum and fecal levels were considered normal for values lower than 48 ng/mL and 107 ng/mL, respectively. I-FABP and DAO serum concentrations were evaluated by ELISA kits (Thermo Fisher Scientific, Waltham, MA, USA) and (Cloud-Clone Corp. Houston, TX, USA), respectively. IL-6 and IL-8 circulating levels were measured by ELISA kits (B.D. Biosciences, Milan, Italy). Lipopolysaccharide (LPS) was assayed using an ELISA kit by Cloud-Clone Corp. (Katy, TX, USA).

### 2.8. Indican and Skatole Evaluation

All patients collected a sample of urine in the morning. A standard colorimetric assay kit (indican assay kit, ABNova Corporation, Taipei, Taiwan) was used according to the manufacturer’s urinary indican determination procedures. The detection and measurement of skatole in urine were performed by the 3-methylindole kit (EurekaLab Division, Chiaravalle, AN, Italy) on a Thermo Scientific model Dionex high-performance liquid chromatography (HPLC) system consisting of an UltiMate 3000 pump and a Rheodyne injector with a 20-μL loop (Sunnyvale, CA, USA). Samples, calibrators, and quality controls were prepared according to the manufacturer’s instructions. In detail, 950 μL of buffer reagent and 20μL of the internal standard were added to 50 μL of a urinary sample. After vortexing, 20 μL of urine samples were injected into the HPLC system. A Poroshell 120 EC-C18 column (2.7 μm, 50 × 4.6 mm; Agilent, Santa Clara, CA, USA) and a mobile phase flow rate of 1.0 mL/min was used for skatole separation. The sample run was 15 min, and spectrofluorimetric detector wavelengths were set at 280 nm (excitation) and 360 nm (emission). Urinary indican and skatole values higher than 20 mg/L and 20 µg/L are considered indices of fermentative and putrefactive dysbiosis, respectively [33].

### 2.9. Statistical Analysis

All results are expressed as means ± SEM unless otherwise specified. Nonparametric tests were performed to avoid violation of the assumption of normal distribution. The Wilcoxon rank-sum test was used to detect differences between the items of the IBS-SSS questionnaire and the biochemical parameters before and after the LFD in the IBS-D patients considered as a whole group or subgroups categorized according to low (L-VD) or normal (N-VD) circulating VD concentrations at the start of the study. The Mann–Whitney test was applied in comparing the two subgroups before and at the end of the diet. Linear regression analysis was performed, considering VD as the dependent variable, and those clinical and biochemical parameters significantly correlated with VD as independent variables in a stepwise regression procedure. The explained variance (adjusted R-squared) was determined for the regression, and it was tested with the F-test. *t*-Values and their significance level were calculated to test the hypothesis that the contribution (the regression coefficient) of an entered variable significantly differed from zero. Pearson’s correlation was performed comparing VD versus all other variables. All the differences were considered significant at a 5% level. A specific statistical package (2005 Stata Statistical software release 9; Stata Corp., College Station, TX, USA) was used.

## 3. Results

### 3.1. Number, Anthropometric Characteristics of the Patients, and Intervention Diet

**Figure 1:**
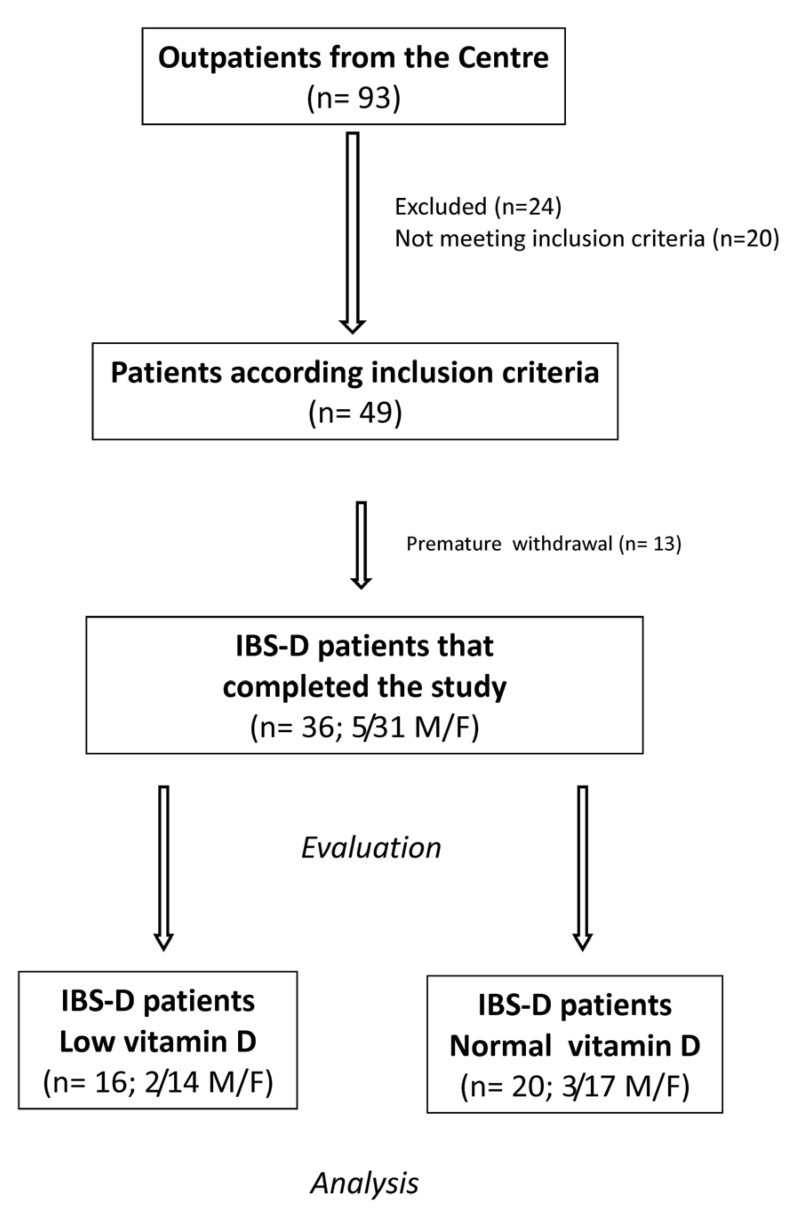
The flow of participants through the study. IBS-D: irritable bowel syndrome with prevalent diarrhea. F: females. M: males.

Figure 1 shows the flow of the patients through the study. Ninety-three, 82 females (F) and 11 males (M), subjects suffering from IBS-D were recruited. Of these patients, 24 were excluded for different reasons, 20 did not meet the inclusion criteria, and 13 declined to participate or were excluded for dietary transgressions. Finally, 36 IBS-D patients (5 men and 31 women; mean age = 43.1 ± 1.7 years) completed the study following LFD for 12 weeks.

The patients’ anthropometric characteristics and serum biochemical parameters at baseline and the end of the diet are summarized in Table 1. Significant decreases (<0.05) in weight, BMI, abdominal and waist circumferences were observed at the end of the diet compared to the start of the study, regardless of the baseline VD status. As for the phosphocalcic metabolism, the serum calcium concentrations were significantly lower (<0.001) and the PTH significantly higher (<0.001) after the LFD compared to baseline, also in this case, regardless of the initial VD circulating concentrations. However, these differences were without clinical impact since all the values were within the normal ranges.

Table 2 shows the main daily nutritional information of patients pre and post LFD, respectively.

Both the protein and carbohydrate percentages significantly increased after LFD in comparison to baseline. By opposite, lipid grams and percentage, as well as the total FODMAP content, significantly decreased at the end of the diet compared to the start of the study. No significant difference in mineral and vitamin levels was observed.

### 3.2. Circulating VD Levels in IBS-D Patients

**Figure 2:**
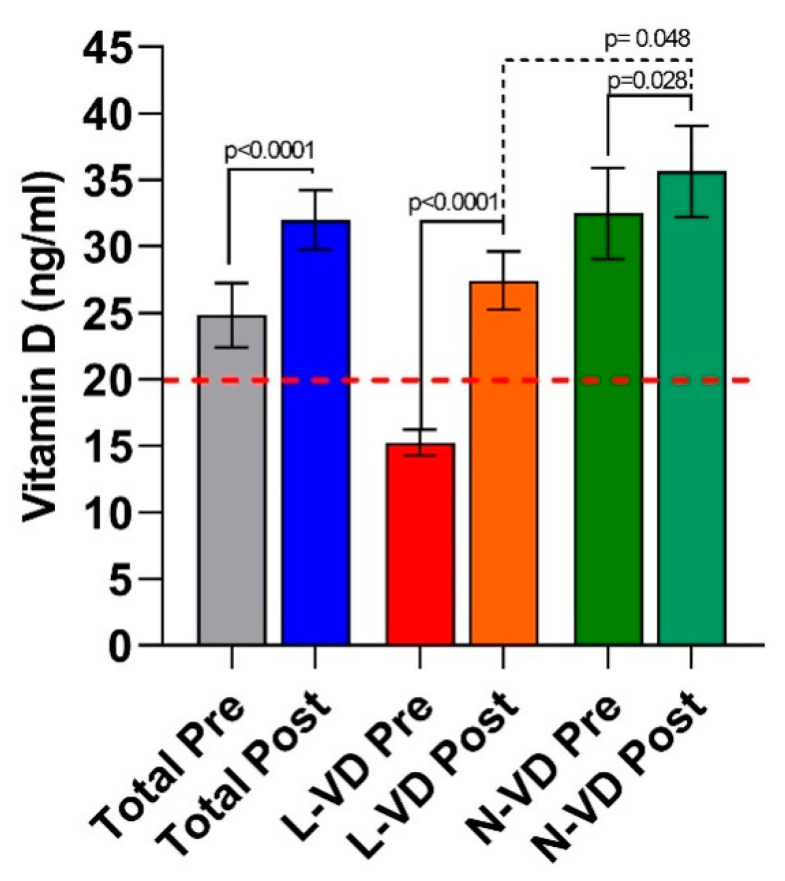
VD levels in IBS-D patients as a whole group and categorized in L-VD and N-VD subgroups according to their low or normal vitamin D (VD) levels at baseline, before (pre) and after (post) 12 weeks of the low FODMAP diet. Data expressed as means ± SEM. Wilcoxon rank-sum test (solid line) was used to compare pre and post-treatment data. The Mann–Whitney test (dotted line) was applied in comparing the two subgroups before and at the end of the diet. Differences considered significant at *p* < 0.05. The red dotted line indicates the cutoff value.

Figure 2 reports the circulating VD levels of the IBS-D patients evaluated at the start and after 12 weeks of LFD.

In the whole group of IBS-D patients, the VD levels increased significantly (*p* < 0.0001) by approximately 28% (25 ± 2.4 vs. 32 ± 2.2 ng/mL) at the end of dietary treatment compared to baseline.

When patients were categorized according to their low or normal VD levels at baseline, 16 out 36 patients (44.4%) had VD levels lower than 20 ng/mL (L-VD); twenty out of 36 patients (55.5%) had normal VD levels, higher than or equal to 20 ng/mL (N-VD).

In the L-VD subgroup, the increase in circulating VD concentrations after the diet (27 ± 2.2 ng/mL) was massive (+80%) and significant (*p* < 0.0001) compared to baseline (15.2 ± 0.98 ng/mL), reaching values indicative of sufficient circulating amounts of the hormone. The N-VD patients also experienced a significant (*p* = 0.028) increase in VD concentrations after LFD (36.2 ± 3.4 ng/mL vs. 32.5 ± 3.4 ng/mL), although at a less extent (+11.4%). Despite the significant rise in VD levels in the L-VD subgroup at the end of LFD, the concentrations of 25(OH)D remained significantly lower (*p* = 0.048) than those in the N-VD patients.

### 3.3. The Symptom Profile in IBS-D Patients

The single and total scores relative to the IBS-SSS questionnaire in the whole group of IBS-D patients are reported in Table 3. The total score decreased significantly by 48.04% after 12 weeks of treatment. When assessing the intervention’s effect on the IBS-SSS items, all of them significantly improved after the diet. In particular, the number of bowel movements per day (“stool frequency”) significantly decreased by 31.6% compared to baseline, while the proportion of patients with a dominant Bristol stool form 5–7 (diarrhea) reduced from 86.1% to 38.9%.

The symptom profiles were then evaluated according to the circulating VD levels at the start of the study. L-VD patients had a significantly worse clinical profile than N-VD patients at the diet’s start. Specifically, the single items relative to intensity and frequency of the “abdominal pain”, “interference on life in general”, “stool frequency”, and the “total score” were all markedly and significantly (*p* < 0.05) higher in the L-VD patients compared to N-VD ones. However, the LFD improved the symptom profile in the totality of IBS-D patients since all the single items and the total score was significantly reduced by 12 weeks of LFD in the two subgroups. More in detail, “abdominal pain” reduced by 49.15% in L-VD and 57.25% in N-VD; “interference on life” reduced by 38.00% in L-VD and 37.92% in N-VD; “stool frequency”, reduced by 34.78% in L-VD and 28.23% in N-VD. Finally, the “total score” was reduced by 45.97% in L-VD and 50.28% in N-VD.

### 3.4. The Small Intestinal Permeability (s-IP)

**Figure 3:**
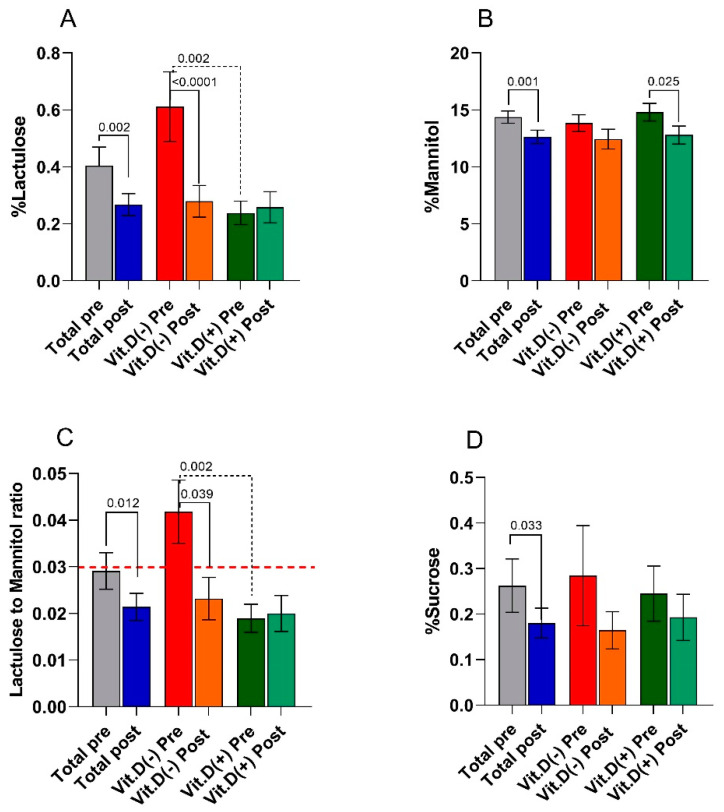
Small intestinal permeability as evaluated by the sugar absorption test (panel (**A**) =% lactulose; panel (**B**) =% mannitol; panel (**C**) = La/Ma ratio; panel (**D**) =% sucrose) before (pre) and after (post) 12 weeks of low FODMAP diet in the IBS-D patients considered as a total group and categorized in L-VD and N-VD subgroups according to their low or normal VD levels at baseline. Data expressed as means ± SEM. Wilcoxon rank-sum test (solid line) was used to compare pre- and post-treatment data. The Mann–Whitney test (black dotted line) was applied in comparing the two subgroups before and at the end of the diet. Differences considered significant at *p* < 0.05. Red dotted line indicates the cutoff value of the La/Ma ratio (0.030).

The s-IP in IBS-D patients was evaluated by the SAT before the diet and the end of treatment. Figure 3 reports the percentage of ingested La (% La), Ma (% Ma), and Su (% Su) evaluated in the urine of the patients. The La/Ma ratio was calculated for each sample.

At the end of the study, in the total IBS-D patients,% La was significantly (*p* = 0.002) lower than the baseline percentages. IBS-D patients after LFD also showed significantly (*p* = 0.001) lower% Ma values than those at the start of the study. Consequently, the La/Ma ratio reduced significantly (*p* = 0.012) by 27.6%. Compared to values at the start of the study, a statistically significant (*p* = 0.033) and sharp (−30.8%) decrease of the excreted sucrose percentage at the end of the diet occurred.

Categorizing the patients according to low or normal basal VD levels at the start of the diet, the Mann–Whitney test revealed a significant difference in% La (*p* = 0.002) and the La/Ma ratio (*p* = 0.002) between the L-VD and N-VD subgroups. Noteworthy, at the start of the study, the L-VD patients, but not the N-VD ones, had a mean La/Ma ratio of 0.042, a value higher than the cutoff (0.030).

After LFD, the nutritional treatment significantly (*p* < 0.0001) reduced by 54% the recovery percentages of lactulose in the L-VD subgroup. N-VD patients showed a significant (*p* = 0.025) reduction by 11.7% in the percentage of mannitol recovery compared to baseline values. In response to LFD, the La/Ma ratio significantly (*p* = 0.039) decreased by 31% in L-VD patients, reaching a value far below the cutoff (0.023) and similar to that in N-VD patients (0.020). Finally, the urinary excretion of sucrose was reduced by LFD in both L-VD (−43%) and N-VD (−24%) groups, although without reaching a statistical significance.

### 3.5. Biomarkers of Intestinal Barrier Function and Integrity

**Figure 4:**
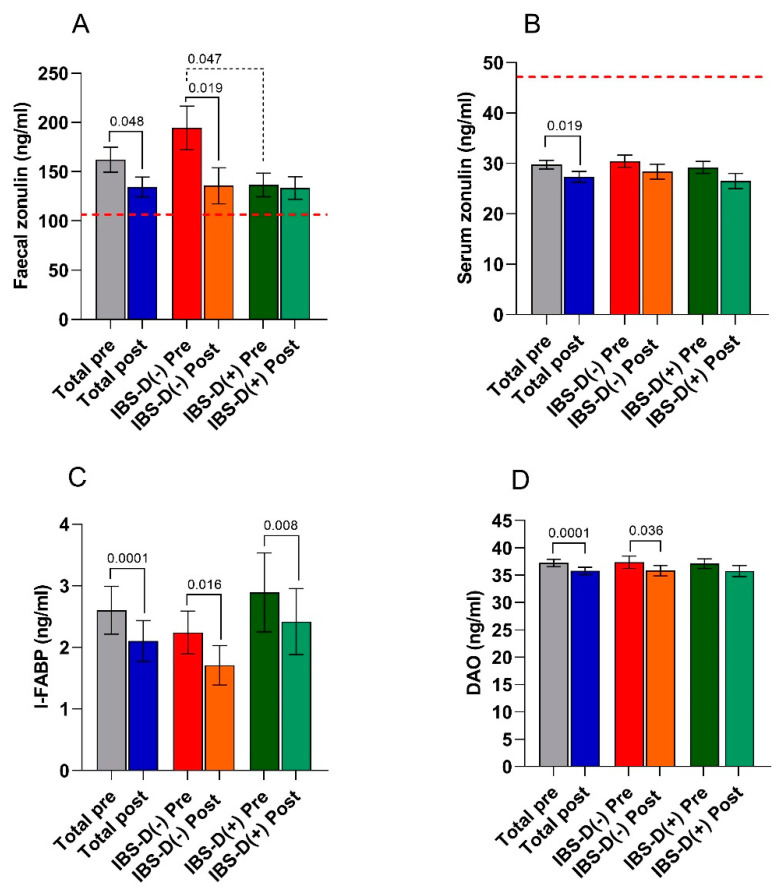
Biomarkers of intestinal barrier function and integrity (panel (**A**) = fecal zonulin; panel (**B**) = serum zonulin, panel (**C**) = intestinal fatty acid-binding protein—I-FABP, and panel (**D**) = diamine oxidase—DAO) before (pre) and after (post) 12 weeks of low FODMAP diet in the IBS-D patients considered as a total group and categorized in L-VD and N-VD subgroups according to their low or normal VD levels at baseline. Data expressed as means ± SEM. Wilcoxon rank-sum test (solid line) was used to compare pre- and post-treatment data. The Mann–Whitney test (black dotted line) was applied in comparing the two subgroups before and at the end of the diet. Differences were considered significant at *p* < 0.05. Red dotted lines indicate the cutoff values for fecal and serum zonulin.

The markers of intestinal barrier function and integrity (fecal and serum zonulin, I-FABP, and DAO) are reported in Figure 4.

The fecal zonulin concentrations of the whole group of IBS-D patients were far above the cutoff level of 107 ng/mL, irrespective of VD levels and the nutritional treatment. As regards the effect of diet, the total group of IBS-D patients had significantly (*p* = 0.048) lower fecal zonulin concentrations (134.4 ± 10.12 ng/mL) than those at the start of the diet (162.22 ± 12.66 ng/mL). In addition, serum zonulin values significantly decreased at the end of the diet (29.75 ± 0.86 ng/mL vs. 27.33 ± 1.06; *p* = 0.019). As for the intestinal barrier integrity, the post-diet concentrations of I-FABP and DAO were significantly (*p* < 0.001) lower than the pre-diet concentrations in the whole IBS-D group.

There was no difference in the values of all the above markers at the start of the diet between L-VD and N-VD subgroups, except for fecal zonulin (194.4 ± 22.1 ng/mL vs. 136.5 ± 11.99 ng/mL; *p* = 0.048, Mann–Whitney test). LFD significantly reduced the levels of fecal zonulin (*p* = 0.019), I-FABP (*p* = 0.016). and DAO (*p* = 0.036) in L-VD patients. Moreover, LFD significantly (*p* = 0.008) reduced only the I-FABP concentrations in the N-VD subgroup.

### 3.6. Indices of Inflammation

The circulating levels of IL-6 and IL-8 in the IBS-D patients are reported in Table 4. In the total group of IBS-D patients, IL-6 and IL-8 levels significantly decreased at the end of the diet (*p* = 0.032 and *p* = 0.019, respectively). No difference in the inflammatory profile was found between NVD and LVD subgroups either before or after the diet.

### 3.7. The Markers of Intestinal Dysbiosis and Bacterial Translocation

**Figure 5:**
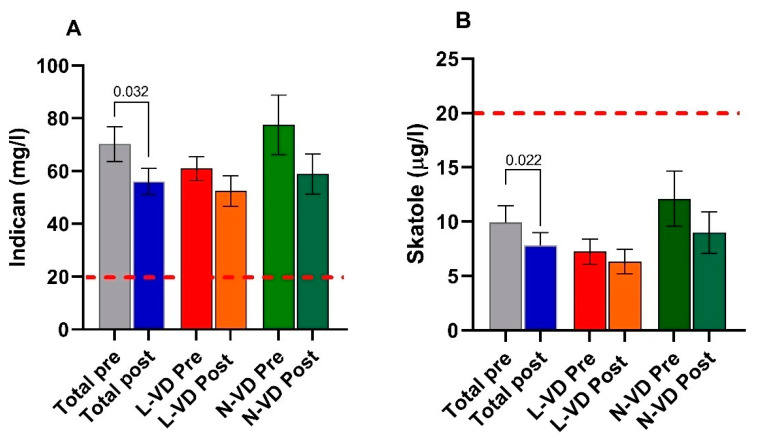
Indican (panel (**A**)) and skatole (panel (**B**)) levels as markers of dysbiosis before (pre) and after (post) 12 weeks of low FODMAP diet in the whole group of IBS-D patients and categorized in L-VD and N-VD subgroups according to their low or normal VD levels at baseline. Data expressed as means ± SEM. Wilcoxon rank-sum-test was used to compare pre- and post-treatment data. The Mann–Whitney test was applied in comparing the two subgroups before and at the end of the diet. Differences considered significant at *p* < 0.05. Red dotted lines indicate the cutoff value.

The indican concentrations in the urine of the whole group of IBS-D patients were far above the cutoff level of 20 mg/mL, irrespective of VD levels and the nutritional treatment. However, a significant decrease (*p* = 0.032) of the indican urinary concentration was observed at the end of LFD in the total IBS-D patients. When the patients were categorized according to VD levels at baseline, no difference was found between N-VD and L-VD. Comparing the indican concentration before and after LFD, no difference was found in both the L-VD subgroup and the N-VD one. Similarly, no difference was found comparing N-VD and L-VD patients at the end of the diet (Figure 5, panel A).

The skatole concentrations in urine were within the limit of the normal range (below 20 µL/L), although a significant decrease was observed after diet in the total group (*p* = 0.022) (Figure 5, panel B).

Finally, in the total group of IBS-D patients, the LPS concentrations were significantly higher (*p* = 0.010) at the start of the study (0.050 ± 0.01 ng/mL) compared to the values at the end of the diet (0.043 ± 0.02 ng/mL). After categorizing for VD levels, L-VD subgroup (0.049 ± 0.002 ng/mL vs. 0.042 ± 0.003 ng/mL), but not the N-VD one (0.044 ± 0.002 ng/mL vs. 0.042 ± 0.02 ng/mL) showed significantly (*p* = 0.042) reduced levels of LPS at the end of the diet compared to baseline.

### 3.8. Correlation and Regression Analyses

Considering all the examinations per each variable, a negative correlation was found between VD levels and IBS-SSS total score (r = −0.34, *p* = 0.004), VD levels and fecal zonulin (r = −0.32, *p* = 0.007). A trend toward significance between VD and La (r = −0.23, *p* = 0.053), and VD and LPS was finally found (r = −0.23, *p* = 0.054). (r = Pearson’s correlation coefficients).

Regression analysis showed that VD levels could be significantly predicted by a linear combination of the variables considered in a stepwise procedure (i.e., IBS-SSS and fecal zonulin) (F = 9.45; df = 2; *p* < 0.001; adjusted R^2^ = 0.19) (Table 5). These data suggest that VD levels may be affected by clinical and biochemical determinants.

## 4. Discussion

Data obtained in this study highlight the close relationship between VD and the intestinal barrier and support the involvement of VD deficiency in the IBS-D pathophysiology. Moreover, the potentially positive role of LFD in the management of IBS-D is further confirmed.

There is massive evidence that the VD deficiency not only has a negative impact on the human skeletal system and the progression of multiple diseases (i.e., cardiovascular diseases, diabetes, autoimmune disease, and neoplasms) but also of gut diseases, including colorectal cancer and IBD [34]. The latter two conditions, and increasing new data in the literature, suggest the importance of low VD circulating levels in the onset of other GI disorders, such as IBS [35].

In our study, at the start of the diet, the whole group of IBS-D patients showed a mean VD concentration only slightly higher than the cutoff value of 20 ng/mL. This vitamin content is now widely accepted as adequate for bone and overall health in healthy individuals [3]. When patients were categorized according to this cutoff value, 44% showed low circulating VD levels. Interestingly, these L-VD patients had higher urinary La excretion, La/Ma ratio, and fecal zonulin concentrations than N-VD patients. These findings suggest the presence of a leaky gut syndrome with a particular failure in the paracellular pathway and support the close relationship between VD and the intestinal barrier [36] as already indicated by in vitro [37], experimental [38], and human studies [39].

From a clinical perspective, L-VD patients also had a significantly higher symptom profile at IBS-SSS than N-VD ones. The VD concentrations appeared to depend on both the symptom profile and the state of health of the intestinal barrier, as linear regression analysis showed. The present findings are in line with the reported association of low circulating VD levels with increased central sensitivity, chronic pain [40], depression [41], and anxiety [42], all determinants related to the risk of IBS development.

As regards the effect of the LFD on IBS, different data in the literature indicate that restricting FODMAP consumption could be beneficial for symptoms and intestinal barrier function [43,44], and a long-term LFD has shown to be more effective than a 30-day diet to control for symptoms fluctuations that occur naturally in all the IBS subtypes irrespective of drug or nutritional treatments. In agreement with this evidence, 12 weeks of LFD significantly reduced symptoms in our IBS-D patients, confirming ours and other previous results [23,45] about its efficacy.

Significant decreases in weight, BMI, abdominal and waist circumferences were observed after LFD. Weight loss and the reduction of BMI were not among the goals of our present study. However, these reductions were probably due to the duration and restrictions of the diet. Weight loss and BMI reduction can represent a possible consequence of a long-term, personalized diet, even if the energy intake during LFD was not significantly reduced. It is known that the “elimination phase” leads to the reduction of body weight and BMI, following the caloric restriction of some foods high in FODMAPs [23].

Previous reports proved that LFD did not modify VD levels in either IBS patients or CD patients treated in combination with a gluten-free diet [46,47]. In the latter study, nutritional adequacy was achieved for zinc and fiber, while not for VD and other micronutrients. Moreover, by conducting a single-blinded randomized trial, Vincenzi et al. [48] reported that IBS patients benefitted from a three-month LFD, which, however, did not modify VD and folic acid levels. By opposite, our results indicate that a long-term LFD significantly improved VD circulating concentrations in IBS-D patients with a significant gain of 28% at the end of the diet. This result may be attributed to the diet per se or better intestinal barrier health conditions that ameliorate the fat absorption in the small intestine. In fact, both s-IP and intestinal barrier integrity significantly improved at the end of the diet. These findings, together with a significant reduction in the markers of inflammation, dysbiosis, and bacterial translocation, may indicate the potentially positive role of a correct diet in the management of IBS-D and support the importance of these factors in its pathophysiology.

Interestingly, when the IBS-D patients were categorized according to the VD status, L-VD patients responded better to diet than N-VD patients. In particular, VD levels increased by 41% in the former group and reached normal values at the end of treatment. While on one side, these results indicate a possible therapeutic role in the control of IBS for VD, on the other, let us hypothesize that dietary components able to alleviate IBS symptoms can also affect VD levels via changes in intestinal permeability. In this context, the s-IP expressed as the urinary La excretion and the La/Ma ratio decreased by 54% and 45%, respectively, in L-VD and IBD-D( + ) at the end of the study, reaching values indicative of a normal s-IP. LFD in L-VD patients concordantly reduced fecal zonulin levels and circulating I-FABP and DAO levels, highlighting improved epithelium integrity. This amelioration resulted in a lower translocation of Gram-negative bacteria throughout the intestinal villi, as evidenced by the lower circulating levels of LPS after diet [49]. On the contrary, and irrespective of VD levels and the diet, the indican concentrations in the urine of the whole group of IBS-D patients were far above the cutoff level of 20 mg/mL, suggesting a massive fermentative dysbiosis. The nutritional treatment alone could probably be insufficient to completely restore the balance in the small intestine’s microbiota.

The present research has some weaknesses. First, VD specific receptor was not evaluated. Thus, we could not obtain complete information about the actual processes occurring at a mucosal level for the vitamin/VDR complex. Second, the cohort of patients was too small to draw firm conclusions. Therefore, further research is needed to investigate the still unveiled relationship between FODMAP, VD levels, and intestinal barrier in IBS. Although our current results and available evidence in the literature support this connection, it is unclear what is causing what. Is VD deficiency causing IBS or, rather, is IBS responsible for VD deficiency? Probably, also other still unknown factors should be taken into account as determinants for both problems. However, based on present and other available data, it seems conceivable that LFD can improve the IBS symptoms by affecting those factors involved in this functional GI disorder, including VD levels and their supposed effects on the integrity and the function of the intestinal barrier. 

## Figures and Tables

**Table 1:** Descriptive statistics of the anthropometric and serum biochemical parameters before (pre) and after (post) 12 weeks of low fermentable oligosaccharides, disaccharides, monosaccharides, and polyols (FODMAP) diet in IBS-D patients as a whole group and categorized according to their low (L-VD) or normal (N-VD) vitamin D levels at baseline.

	Total Pre (*n* = 36)	Total Post (*n* = 36)	Rank-Sum Test *p*	L-VD Pre (*n* = 16)	L-VD Post (*n* = 16)	Rank-Sum Test *p*	N-VD Pre (*n* = 20)	N-VD Post (*n* = 20)	Rank-Sum Test *p*
Age (Years)	43.10 ± 1.70	//	//	43.90 ± 2.90	//	//	42.50 ± 2.00	//	//
Height (cm)	1.60 ± 0.01	//	//	1.60 ± 0.10	//	//	1.60 ± 0.020	//	//
Weight (kg)	65.50 ± 2.10	62.00 ± 1.90	<0.001	66.70 ± 3.70	63.50 ± 3.60	<0.001	64.60 ± 2.30	60.80 ± 2.10	<0.001
BMI (kg/m^2^)	25.10 ± 0.80	23.80 ± 0.80	<0.001	25.90 ± 1.40	24.70 ± 1.40	<0.001	24.50 ± 0.80	23.20 ± 0.80	<0.001
Abdominal circumference (cm)	88.90 ± 1.80	86.20 ± 1.80	<0.001	89.10 ± 3.30	86.40 ± 3.20	0.034	88.90 ± 1.90	86.00 ± 2.10	<0.001
Waist circumference (cm)	79.10 ± 2.00	76.30 ± 1.90	<0.001	79.80 ± 3.80	77.70 ± 3.50	0.011	78.60 ± 2.20	75.30 ± 1.90	<0.001
Creatinine (mg/dL)	0.76 ± 0.04	0.75 ± 0.04	0.85	0.73 ± 0.05	0.74 ± 0.05	0.68	0.78 ± 0.05	0.76 ± 0.05	0.63
Urea (mg/dL)	32.64 ± 1.38	33.91 ± 1.20	0.31	33.25 ± 2.15	33.87 ± 1.50	0.93	32.16 ± 1.83	33.84 ± 1.75	0.33
Calcium (mg/dL)	9.42 ± 0.07	8.84 ± 0.06	<0.001	9.46 ± 0.10	8.84 ± 0.07	<0.001	9.39 ± 0.10	8.84 ± 0.10	<0.001
Phosphorous (mg/dL)	3.64 ± 0.13	3.65 ± 0.12	0.83	3.45 ± 0.19	3.45 ± 0.18	0.79	3.79 ± 0.17	3.80 ± 0.16	0.95
PTH (pg/mL)	42.37 ± 2.31	42.85 ± 2.32	<0.001	43.45 ± 3.30	43.93 ± 3.33	<0.001	41.50 ± 3.28	41.99 ± 3.28	<0.001

BMI: body mass index. PTH: parathyroid hormone. Data expressed as means ± SEM. Wilcoxon signed-rank test was used to compare pre- and post-treatment data. The Mann–Whitney test was applied in comparing the two subgroups before and at the end of the diet. Differences considered significant at *p* < 0.05; n.s. = not significant.

**Table 2:** Energy, basal metabolism, and nutrient intake of IBS-D subjects before (pre) and after (post) 12 weeks of low FODMAP.

	Total Pre (*n* = 36)	Total Post (*n* = 36)	*p*
Energy consumption (kcal)	2062 ± 68.54	2058 ± 67.55	0.967
Energy intake (kcal)	2054 ± 129.30	1811 ± 92.22	0.058
Basal metabolism (kcal)	1485 ± 29.86	1502 ± 32.80	0.056
Proteins (g)	78.81 ± 4.60	86.81 ± 3.97	0.783
Proteins (%)	15.91 ± 0.31	19.50 ± 0.16	<0.0001
Lipids (g)	87.28 ± 7.36	59.09 ± 3.12	0.0011
Lipids (%)	36.35 ± 0.88	29.67 ± 0.17	<0.0001
Carbohydrates (g)	236.40 ± 11.75	242.40 ± 12.94	0.237
Carbohydrates (%)	47.24 ± 0.88	50.54 ± 0.24	0.002
Alcohol (%)	0.74 ± 0.21	0.29 ± 0.12	0.178
Dietary fiber (g)	18.05 ± 0.76	16.97 ± 0.92	0.060
Total FODMAPs (g/day)	20.71 ± 0.80	3.26 ± 0.08	<0.0001
Calcium (mg)	813.80 ± 6.17	789.10 ± 11.18	0.061
Chloride (mg)	3.97 ± 0.03	3.89 ± 0.06	0.118
Copper (mg)	1.30 ± 0.01	1.25 ± 0.02	0.104
Iodine (µg)	117.7 ± 0.98	122.6 ± 2.81	0.056
Iron (mg)	11.54 ± 0.07	10.94 ± 0.27	0.064
Magnesium (mg)	279.3 ± 1.87	275.6 ± 2.13	0.072
Potassium (mg)	2.82 ± 0.01	2.87 ± 0.04	0.133
Phosphorous (mg)	1.25 ± 0.01	1.24 ± 0.01	0.064
Selenium (µg)	45.31 ± 0.40	47.17 ± 1.05	0.119
Sodium (mg)	2.74 ± 0.01	2.68 ± 0.05	0.120
Zinc (mg)	8.51 ± 0.05	8.34 ± 0.07	0.060
Vitamin A (µg)	1.04 ± 0.03	1.11 ± 0.03	0.089
Vitamin B6 (mg)	1.92 ± 0.05	2.01 ± 0.02	0.217
Vitamin B12 (µg)	5.14 ± 0.12	5.47 ± 0.11	0.074
Vitamin C (mg)	95.64 ± 1.54	98.50 ± 1.70	0.070
Niacin (mg)	19.98 ± 0.13	20.46 ± 0.20	0.060
Riboflavin (mg)	1.51 ± 0.01	1.45 ± 0.02	0.077
Thiamin (mg)	1.50 ± 0.02	1.44 ± 0.02	0.078
Folate (µg)	231.60 ± 1.99	227.0 ± 2.30	0.118

Data expressed as means ± SEM; *p*-value was determined by Wilcoxon signed-rank test; differences considered significant at *p* < 0.05.

**Table 3:** Irritable bowel syndrome symptom severity scale (IBS-SSS) (IBS)-SSS single items and total score before (pre) and after (post) 12 weeks of low FODMAP diet in IBS-D patients as a total group and categorized according to their low (L-VD) or normal (N-VD) vitamin D levels at baseline.

	Total Pre (*n* = 36)	Total Post (*n* = 36)	Rank-Sum Test *p*	L-VD Pre (*n* = 16)	L-VD Post (*n* = 16)	Rank-Sum Test *p*	N-VD Pre (*n* = 20)	N-VD Post (*n* = 20)	Rank-Sum Test *p*
Abdominal pain intensity	48.3 ±3.7	22.9 ± 3.5	<0.001	59.0 ± 4.6 ^a^	30.0 ± 6.3	0.003	40.0 ± 4.8 ^a^	17.1 ± 3.4	<0.001
Abdominal pain frequency	43.3 ± 4.5	22.6 ± 4.2	<0.001	58.0 ± 6.2 ^b^	26.9 ± 7.2	0.002	38.0 ± 5.5 ^b^	19.1 ± 5.0	0.014
Abdominal distension	55.9 ± 3.7	26.3 ± 3.6	<0.001	54.2 ± 6.7	28.7 ± 5.8	0.005	57.1 ± 4.2	24.4 ± 4.5	<0.001
Dissatisfaction of bowel habit	67.8 ± 3.7	35,7 ± 3.9	<0.001	72.3 ± 4.3	40.4 ± 6.3	0.002	64.2 ± 5.8	31.8 ± 4.8	<0.001
Interference on life in general	57.8 ± 3.2	34.5 ± 4.2	<0.001	65.0 ± 4.4 ^c^	40.3 ± 6.8	0.005	48.0 ± 5.2 ^c^	29.8 ± 5.1	0.003
Stool frequency	1.9 ± 0.2	1.3 ± 0.1	<0.001	2.3 ± 0.2 ^d^	1.5 ± 0.2	0.014	1.7 ± 0.2 ^d^	1.22 ± 0.2	0.008
Total score	273.1 ± 12.6	141.9 ± 15.1	<0.001	308.0 ± 14.0 ^e^	166.4 ± 25.5	<0.001	246.0 ± 15.0 ^e^	122.3 ± 17.2	<0.001

Data are expressed as means ± SEM. Wilcoxon signed-rank test was used to compare pre- and post-treatment data. The Mann–Whitney test was applied in comparing the two subgroups before and at the end of the diet. Superscript letters in brackets indicate significant differences between L-VD and N-VD patients before starting the study. ^a^ *p* = 0.0122; ^b^ *p* = 0.0334; ^c^ *p* = 0.0134; ^d^ *p* = 0.0259; ^e^ *p* = 0.008. No differences were found comparing L-VD and N-VD patients after diet. All differences considered significant at *p* < 0.05.

**Table 4:** Circulating levels of IL-6 and IL-8 before (pre) and after (post) 12 weeks of low FODMAP diet in IBS-D patients categorized in L-VD and N-VD subgroups according to their low or normal VD levels at baseline.

	Total Pre (*n* = 36)	Total Post (*n* = 36)	Rank-Sum Test *p*	L-VD Pre (*n* = 16)	L-VD Post (*n* = 16)	Rank-Sum Test *p*	N-VD Pre (*n* = 20)	N-VD Post (*n* = 20)	Rank-Sum Test *p*
IL-6 (pg/mL)	5.3 ± 0.13	5.1 ± 0.13	0.020	5.4 ± 0.24	5.1 ± 0.21	0.232	5.3 ± 0.2	5.1 ± 0.2	0.052
IL-8 (pg/mL)	4.5 ± 0.40	4.0 ± 0.16	0.019	4.0 ± 0.18	3.8 ± 0.14	0.109	4.9 ± 0.71	4.2 ± 0.26	0.113

Data expressed as means ± SEM. Wilcoxon rank-sum-test was used to compare pre- and post-treatment data. The Mann–Whitney test was applied in comparing the two subgroups before and at the end of the diet. Differences considered significant at *p* < 0.05.

**Table 5:** Regression analysis between VD levels and clinical and biochemical variables.

Parameters	β	Std. Error (β)	*p*	95% CI
IBS-SSS tot	−0.047	0.015	0.002	−0.075–−0.018
Fecal zonulin	−0.065	0.022	0.004	−0.108–−0.022

Linear regression analysis was performed considering the VD levels as the dependent variable and the other parameters as independent variables. IBS-SSS = irritable bowel syndrome symptom scoring system.

## Data Availability

The datasets used and/or analyzed during the current study are available from the corresponding author on reasonable request.
